# Supplementary material for: Pruriception and neuronal coding in nociceptor subtypes in human and nonhuman primates
Source: eLife. 2021 Apr 23;10:e64506. doi: 10.7554/eLife.64506 (PMC8064749; doi:10.7554/eLife.64506)
Supplement: Supplementary file 1. — The number of single- and double-positive neurons is given as aggregated number for each DRG. [file elife-64506-supp1.docx]

**Supplementary File 1**

Expression of MRGPRD and MRGPRX1 was assessed in three macaque DRGs, using double-labeling ISH. The number of single and double-positive neurons is given as aggregated number for each DRG.

|  | **MRGPRD^+^ neurons** | **MRGPRX1^+^ neurons** | **MRGPRX1+D^+^ neurons** |
| --- | --- | --- | --- |
| **DRG 1**  **(4 slices)** | 17 | 203 | 135 |
| **DRG 2**  **(2 slices)** | 13 | 41 | 45 |
| **DRG 3**  **(1 slice)** | 9 | 5 | 19 |
| **Totals** | 39 | 249 | 199 |
