# Supplementary material for: Pruriception and neuronal coding in nociceptor subtypes in human and nonhuman primates
Source: eLife. 2021 Apr 23;10:e64506. doi: 10.7554/eLife.64506 (PMC8064749; doi:10.7554/eLife.64506)
Supplement: Supplementary file 2. — *Bonferroni post hoc p-values compared to HIS. **Bonferroni post hoc p-values compared to BAM8-22 + ALA + HIS. Units are in cm2 and calculated as mean ± standard error of the mean (SEM). Data from all 29 subjects were included in the analysis. [file elife-64506-supp2.docx]

**Supplementary File2.**

Statistical analysis of areas (cm^2^) of alloknesis, hyperalgesia, hyperknesis, wheal and flare/ or local erythema evoked by injection of a BAM8-22, ALA, HIS, a combination of BAM8-22 and ALA, and a combination of BAM8-22 and ALA and HIS.

|  | **BAM8-22** | **ALA** | **HIS (*)** | **BAM8-22 + ALA** | **BAM8-22 + ALA + HIS (**)** |
| --- | --- | --- | --- | --- | --- |
| **Alloknesis** | 4.0 ± 1.2  *p =0.000043  **p = 0.04701 | 3.7 ± 0.9  *p = 0.000024  **p = 0.03076 | 17.4 ± 3.8 | 3.7 ±1.4  *p = 0.00002769  **p = 0.03387 | 12.0 ± 2.9 |
| **Hyperalgesia** | 8.7 ±2.1  *p = 0.00009063  **p = 0.001371 | 5.7 ± 1.4  *p = 0.0000006871  **p = 0.00001542 | 21.0 ± 3.8 | 10.6 ± 2.5  *p = 0.001512  **p = 0.01673 | 19.1 ± 3.5 |
| **Hyperknesis** | 7.2 ± 1.9  *p = 0.01417  **p = 0.007371 | 5.4 ± 1.5  *p = 0.001226  **p = 0.0005838 | 15.4 ± 2.9 | 10.0 ± 2.6 | 15.9 ± 3.2 |
| **Wheal** | 0.5 ± 0.1  *p < 0.0000001  **p < 0.0000001 | 0.3 ± 0.0  *p < 0.0000001  **p < 0.0000001 | 1.9 ± 0.1 | 0.6 ± 0.1  *p < 0.0000001  **p < 0.0000001 | 1.9 ± 0.2 |
| **Flare/Erythema** | 5.4 ± 1.5  *p < 0.0000001  **p < 0.0000001 | 1.2 ± 0.7  *p < 0.0000001  **p < 0.0000001 | 21.5 ± 3.0 | 5.7 ± 1.0  *p < 0.0000001  **p < 0.0000001 | 21.0 ± 2.6 |

*: Bonferroni post hoc p-values compared to HIS. **: Bonferroni post hoc p-values compared to BAM8-22 + ALA + HIS. Units are in cm^2^ and calculated as mean ± SEM. Data from all 29 subjects were included in the analysis.
