# Supplementary material for: Pruriception and neuronal coding in nociceptor subtypes in human and nonhuman primates
Source: eLife. 2021 Apr 23;10:e64506. doi: 10.7554/eLife.64506 (PMC8064749; doi:10.7554/eLife.64506)
Supplement: Supplementary file 3. — EnsemblGeneIDs, gene names, and the species of the gene are shown. [file elife-64506-supp3.docx]

**Supplementary File 3**

Protein sequences used for phylogenetic tree construction were fetched from Ensembl. Ensembl Gene IDs, gene names and the species of the gene are shown.

| **EnsemblGeneID** | **Name** | **Species** |
| --- | --- | --- |
| ENSG00000170255 | MRGPRX1 | Human |
| ENSG00000172938 | MRGPD | Human |
| ENSMMUG00000060151 | MRGPRX1 | Macaque |
| NSMMUG00000010129 | MRGPRD | Macaque |
| ENSMUSG00000078698 | Mrgpra3 | Mouse |
| ENSMUSG00000070552 | Mrgprc | Mouse |
| ENSMUSG00000051207 | Mrgprd | Mouse |
| ENSRNOG00000014227 | Mrgpra | Rat |
| ENSRNOG00000014242 | Mrgprc | Rat |
| ENSRNOG00000013448 | Mrgprd | Rat |
