# Supplementary material for: Pruriception and neuronal coding in nociceptor subtypes in human and nonhuman primates
Source: eLife. 2021 Apr 23;10:e64506. doi: 10.7554/eLife.64506 (PMC8064749; doi:10.7554/eLife.64506)
Supplement: Supplementary file 4. — DRG tissue was obtained from Tissue For Research (donors 1 and 2) and the NIH NeuroBioBank (donors 3 and 4). Basic clinical data for each donor are summarized. [file elife-64506-supp4.docx]

**Supplementary File 4: Clinical data for human DRG donors.**

DRG tissue was obtained from Tissue For Research (donor 1 and 2) and the NIH NeuroBioBank (donor 3 and 4). Basic clinical data for each donor are summarized.

| donor ID | age | gender | race | cause leading to death |
| --- | --- | --- | --- | --- |
| 1 | 42 | female | white | chronic inflammatory demyelinating polyneuritis |
| 2 | 68 | male | white | chronic obstructive pulmonary disease |
| 3 | 48 | male | black | congestive heart failure |
| 4 | 41 | male | white | atherosclerosis |
